# Selective catalytic reductive amination of furfural to furfurylamine: advances in catalyst design strategies and mechanistic insights

**DOI:** 10.1039/d6ra02687g

**Published:** 2026-07-06

**Authors:** Hao Zhou, Qin Liu, Zhixin Jia, Xuejin Zhang

**Affiliations:** a School of Environmental and Nature Resources, Zhejiang University of Science and Technology Hangzhou Zhejiang 310023 P. R. China haozhou@zust.edu.cn; b International College, Zhejiang University of Technology Hangzhou Zhejiang 310023 P. R. China

## Abstract

The efficient transformation of biomass platform molecules into high-value-added N-containing chemicals is a central focus of sustainable chemistry. The reductive amination of furfural (FAL) to furfurylamine (FAM) represents a highly attractive route. However, this reaction involves a complex network with multiple parallel and consecutive pathways, including direct hydrogenation, over-amination, furan ring saturation, and trimerization, posing significant challenges for catalyst selectivity. This review comprehensively summarizes recent advances in heterogeneous catalysts for the selective reductive amination of FAL to FAM. Catalytic systems are categorized and discussed based on their primary action mechanisms: (1) electronic modulation strategies (*e.g.*, constructing electron-deficient or electron-rich metal centers); (2) metal–support interactions (MSI) and interface engineering (*e.g.*, utilizing reducible supports, constructing hydrogen spillover pathways); (3) precise control of active site structure (*e.g.*, morphology control, single-atom/alloy catalysts); and (4) acid–base site synergistic catalysis (*e.g.*, cooperation between Lewis/Brønsted acid sites and metal sites). Through an in-depth analysis of the structure–activity relationships in each category, this review aims to provide theoretical guidance for the rational design of high-performance reductive amination catalysts. Finally, future research directions are outlined, including enhancing catalyst stability, *in situ*/*operando* mechanistic studies, innovation in low-ammonia/ammonia-free systems, process intensification, and expansion to other biomass substrates.

## Abstract 2

The efficient transformation of biomass platform molecules into high-value-added N-containing chemicals is a central focus of sustainable chemistry.

## Graphical abstract

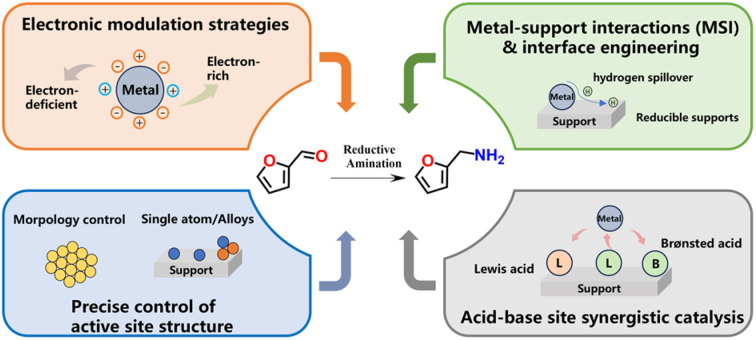

## 1. Introduction

The depletion of fossil resources and growing environmental concerns have intensified global efforts towards producing high-value chemicals from renewable biomass.^1–7^ Furfural (FAL), a key platform molecule derived primarily from the hydrolysis and dehydration of hemicellulose in agricultural and forestry waste, holds significant importance for valorizing lignocellulosic biomass.^8–13^ Among various transformation pathways for FAL, reductive amination to produce furfurylamine (FAM) is particularly attractive due to its atom economy and short synthetic route.^14–17^ FAM is a valuable primary amine with wide applications in pharmaceuticals, agrochemicals, polymers, and surfactants. For instance, it serves as a key intermediate for the side chain of the antimalarial drug chloroquine phosphate and is used in producing high-performance epoxy resin hardeners, corrosion inhibitors, and specialty polymers.^18–23^

**Scheme 1:**
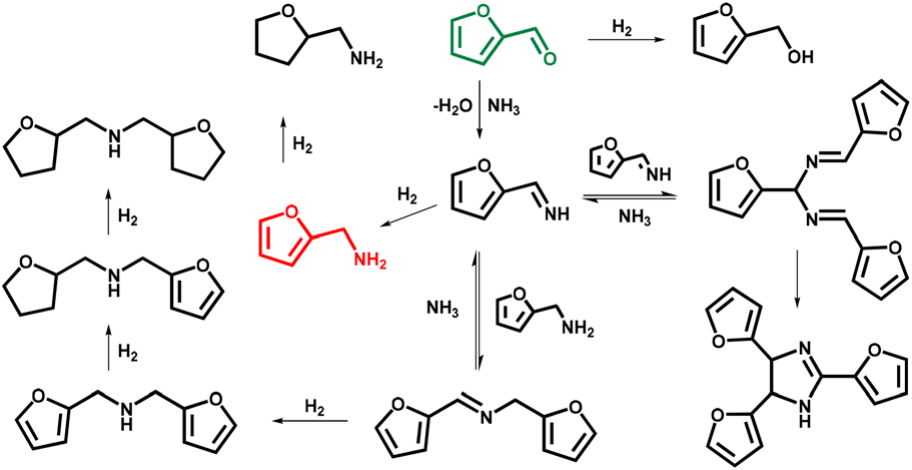
Reaction network for reductive amination of furfural to furfurylamine.

Despite being thermodynamically favorable, the reductive amination of FAL to FAM involves a complex reaction network (Scheme 1), presenting significant selectivity challenges: (1) the nucleophilic nature of FAM can lead to further reactions with intermediates (*e.g.*, imines), generating secondary amines, tertiary amines, or trimers as by-products. Thermodynamically, forming secondary amines *via* intermediate condensation is highly exergonic and more favorable than stopping at the primary amine stage (Table 1). (2) The aldehyde group of FAL may undergo direct hydrogenation to furfuryl alcohol (FOL), reducing the yield of the target product. (3) Undesirable saturation of the C=C bonds in the furan ring can occur, destroying the unique conjugated structure and affecting downstream product properties. (4) Polymerization or rearrangement of intermediates or products may happen under high temperatures or acidic conditions. Consequently, developing heterogeneous catalysts with high activity, selectivity, and stability is the core challenge in this field.

**Table 1:** Representative standard Gibbs free energy changes for the primary amination and secondary condensation pathways during the reductive amination of 5-hydroxymethylfurfural^24^

Reaction pathway	Computational free energy (kJ mol^−1^)
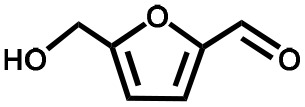	0
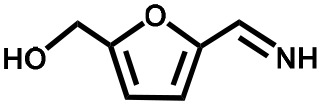	26.2
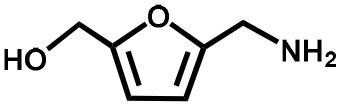	−44.9
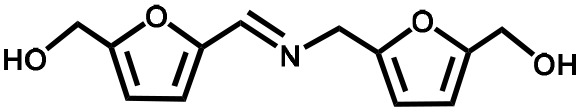	20.3
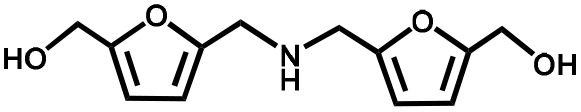	−38.0

Traditional noble metal catalysts (*e.g.*, Pt, Pd, Ru) demonstrate considerable activity owing to their excellent hydrogenation capability and ability to facilitate C–N bond formation.^25–34^ However, their high cost and scarcity limit large-scale industrial application. Recently, non-noble metal catalysts (*e.g.*, Ni, Co) and low-loading, high-performance noble metal catalysts through surface nano-structuring have become research priorities.^35–41^ Successful catalyst design has evolved from merely pursuing high activity to a deeper understanding of reaction mechanisms and the precise modulation of the electronic structure, geometric structure, and micro-environment of active sites.

This review aims to systematically summarize recent progress in heterogeneous catalysis for the selective reductive amination of FAL to FAM. Distinct from previous reviews, it focuses on categorizing and evaluating catalytic systems based on their primary action mechanisms, delving into the influence of key factors such as electronic effects, metal–support interactions, active site structure, and acid–base synergy on catalytic performance (activity, selectivity, stability). By analyzing structure–activity relationships, it provides theoretical guidance for the rational design of high-performance reductive amination catalysts. Finally, challenges and future research directions are discussed to advance the green synthesis of biomass-derived N-containing chemicals.

## 2. Electronic structure modulation strategies

The modulation of the electron density of active metal centers is a highly effective strategy for optimizing their hydrogenation activity and selectivity. By altering the electronic properties of metal nanoparticles, the adsorption strength of reactants, intermediates, and products can be tuned, thereby changing reaction pathways and energy barriers to achieve precise selectivity control.

**Fig. 1:**
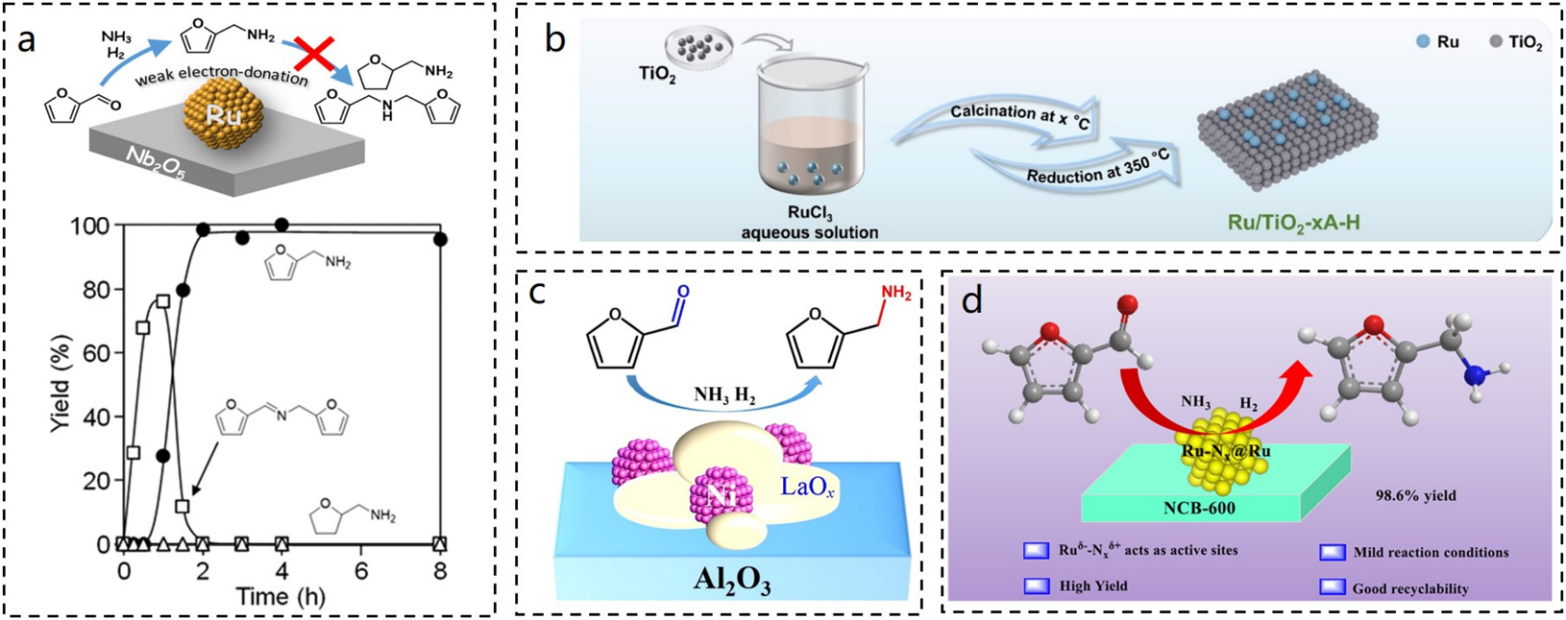
Electronic structure modulation strategies on reductive amination of furfural. (a) Weak electron-donating capability of Ru particles on the Nb_2_O_5_ surface.^42^ Reproduced from ref. 42 with permission from American Chemical Society, copyright 2017. (b) Electron-deficient Ru^0^ and lewis acid sites over Ru/TiO_2_-200A-H catalyst.^43^ Reproduced from ref. 43 with permission from American Chemical Society, copyright 2025. (c) Electron-deficient Ni^0^ species induced by addition of LaO_*x*_.^44^ Reproduced from ref. 44 with permission from Springer Nature, copyright 2023. (d) Electron-rich Ru catalyst constructed by doping N into coffee biochar.^45^ Reproduced from ref. 45 with permission from American Chemical Society, copyright 2024.

The creation of electron-deficient centers by supporting metals on supports with electron-withdrawing properties or through interaction with more electronegative components can lower the electron density of the metal. The electron-deficient state has been demonstrated to be particularly effective in suppressing undesired side reactions, such as over-hydrogenation. Komanoya *et al.* reported the Ru/Nb_2_O_5_ catalyst system for reductive amination of furfural.^42^ Nb_2_O_5_, despite its status as a weak electron donor, interacts with Ru nanoparticles, leading to Ru sites characterized by relatively low electron density. These electron-deficient Ru sites favor the adsorption and hydrogenation of imine intermediates while significantly inhibiting the over-hydrogenation of the furan ring, achieving a 99% FAM yield at 363 K and 4 MPa H_2_ (Fig. 1a). Mechanistic studies suggest that the Ru sites with low electron density alter the reactivity of surface hydrogen species, rendering them more likely to attack the C=N bond in the imine rather than the C=C bond in the furan ring. Similarly, in the Ru/TiO_2_ catalyst system,^43^ the modulation of the electron density of Ru^0^ and the content of RuO_*x*_ species is achieved through the control of the calcination temperature. The Ru/TiO_2_-200A-H catalyst with the calcination temperature of 200 °C, possesses electron-deficient Ru^0^ and the highest density of Lewis acid sites derived from RuO_*x*_ (Fig. 1b). This unique electronic and acidic synergy enables efficient catalysis even at a low temperature of 30 °C, with a FAM yield of 97.8%. Kinetic analysis revealed that electron-deficient Ru^0^ enhances activation of H_2_, while Lewis acid sites (RuO_*x*_) promote the adsorption and activation of NH_3_, FAL, and Schiff bases. Their cooperation significantly reduces the apparent activation energy (16.8 kJ mol^−1^) for the ammonolysis of the Schiff base, which is identified as the rate-determining step. Introducing lanthanum (La) is a classical strategy for modulating electronic structure. In the reported Ni/Al_2_O_3_–LaO_*x*_ catalysts,^44^ the addition of La induces electron transfer from Ni to LaO_*x*_ species, forming electron-deficient Ni^0^ active centers. The electron-deficient Ni species facilitate the activation of N-containing intermediates, while the smaller Ni particle size (resulting from La addition) suppresses the direct hydrogenation of FAL to furfuryl alcohol (Fig. 1c). The optimal catalyst (Ni/Al_2_O_3_–0.5LaO_*x*_) achieved a 94.9% FAM yield with excellent stability under mild conditions (90 °C, 2 MPa H_2_, 2 MPa NH_3_). The promotion effect of La is multifaceted: it modulates the electronic state of Ni, refines Ni nanoparticles, increases the number of surface strong acid sites, and enhances H_2_ dissociation and spillover capability.

Conversely, increasing the electron density of metal centers, for example, through nitrogen doping, to form electron-rich metal centers can also enhance catalytic performance in certain cases, particularly regarding activation of H_2_. Ru/NCB-600 (ruthenium supported on N-doped coffee biochar) is a prime example.^45^ In this system, the interaction between the N-doped coffee biochar (NCB) and Ru leads to the formation of a Ru^*δ*−^–N_*x*_^*δ*+^ structure, creating electron-rich Ru species. This special structure act as an efficient Frustrated Lewis Pair (FLP),^46,47^ promoting the heterolytic cleavage of H_2_ and significantly lowering the reaction energy barrier. 98.6% FAM yield could be obtained with a formation rate of 95.6 g_FAM_ g_Ru_^−1^ h^−1^ at 50 °C and 2 MPa H_2_ (Fig. 1d). Kinetic experiments confirmed that its apparent activation energy (23.0 kJ mol^−1^) was significantly lower than that of an undoped reference catalyst (58.8 kJ mol^−1^), highlighting the profound impact of electronic modulation on reaction kinetics. The synergistic interface between metallic Co^0^ and oxygen-deficient CoO_*x*_ in Co@CoO_*x*_ catalysts also exemplifies how specific electronic structures promote H_2_ dissociation and C=N bond activation.^48^ The CoO_*x*_-250 catalyst, obtained by reduction at 250 °C, exhibited an optimal Co^0^/CoO_*x*_ ratio and abundant oxygen vacancies. This structure not only facilitated the heterolytic dissociation of H_2_ but also activated the C=N bond *via* oxygen vacancies. Their synergistic effect achieved a 96.4% FAM yield at 60 °C and 3 MPa H_2_, maintaining high activity (95.8%) even at 30 °C.

The successful implementation of electronic modulation strategies requires balancing the hydrogenation capability and selectivity of the metal center. While electron-deficient centers typically help to suppress over-hydrogenation, excessive electron deficiency may lead to insufficient hydrogenation activity. Electron-rich centers can enhance activation of H_2_ but might promote over-hydrogenation. Therefore, the electronic density of the metal must be precisely designed according to the target reaction pathway.

## 3. Metal–support interactions (MSI) and interface engineering

The interaction between metal nanoparticles and the support (metal–support interaction, MSI) is a foundational concept in heterogeneous catalysis. The rational design of support properties and metal–support interface structures has been demonstrated to significantly modify the geometric and electronic properties of catalysts, thereby influencing their catalytic behavior. Using reducible metal oxides (*e.g.*, TiO_2_, MoO_3_, Nb_2_O_5_) as supports result in forming of oxygen vacancies^49^ and specific interface structures under reducing conditions, inducing strong metal–support interaction (SMSI), which profoundly affects catalytic performance.^50–57^

**Fig. 2:**
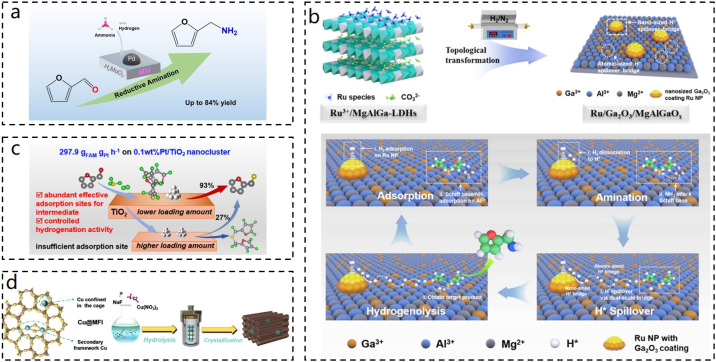
Metal–support interactions and interface engineering on reductive amination of furfural. (a) Metal–support interactions on the Pd/MoO_3−*x*_ catalyst.^58^ Reproduced from ref. 58 with permission from John Wiley and Sons, copyright 2023. (b) Dual scale hydrogen transfer bridge construction on the Ru/Ga_2_O_3_/MgAlGaO_*x*_ catalyst.^60^ Reproduced from ref. 60 with permission from American Chemical Society, copyright 2023. (c) Nanocluster proximity effect on the Pt/TiO_2_ catalyst.^59^ Reproduced from ref. 59 with permission from American Chemical Society, copyright 2025. (d) Accurate restricted transition-state shape on the Cu@MFI catalyst.^61^ Reproduced from ref. 61 with permission from Elsevier, copyright 2025.

Wang *et al.* reported the Pd/MoO_3−*x*_ catalysts rich in Mo^5+^ species by adjusting the reduction temperature from 300 to 600 °C.^58^ Characterization results indicated that Mo^5+^ species act as Lewis acid sites promoting carbonyl activation. Simultaneously, the strong metal–support interaction modulated the electronic structure of Pd, favoring the hydrogenolysis of Schiff base (N-furfurylidenefurfurylamine) and its germinal diamine intermediate to form the target product. Pd/MoO_3−*x*_-500 catalyst achieved the 84% FAM yield at 80 °C and 2 MPa H_2_, significantly outperforming Pd catalysts on conventional supports like SiO_2_ or Al_2_O_5_. Time-course studies and characterization (XPS, NH_3_-TPD, H_2_-TPD) confirmed the crucial role of the Pd-H_*x*_MoO_3_ interface in the reaction pathway (Fig. 2a). The “nanocluster proximity effect” in Pt/TiO_2_ catalysts is another excellent example of interface engineering.^59^ A “top-down” method involving the calcination of PVP-protected Pt nanoparticles on TiO_2_ at 550 °C in air led to their redispersion into ∼1 nm Pt nanoclusters. It was found that reducing the Pt loading (*e.g.*, to 0.1 wt%) increased the distance between Pt clusters, thereby increasing the number of Ti^4+^ sites adjacent to Pt. This proximity promoted the adsorption and conversion of the key Schiff base intermediate while inhibiting hydrogen spillover intensity and avoiding over-hydrogenation. The FAM yield exceeded 93% was achieved at 100 °C with 0.5 MPa NH_3_, and 2 MPa H_2_ (Fig. 2c). DFT calculations revealed a competitive Langmuir–Hinshelwood mechanism where NH_3_ and H_2_ compete for adsorption on Pt sites, with NH_3_ preferentially participating in the ammonolysis of the Schiff base to form the primary amine.

The adsorption/desorption dynamics of the FAM on transition metal surfaces strongly dictate the chemoselectivity. Transition metals (*e.g.*, Pt, Pd, Ni, and Ru) possess unfilled d-orbitals that readily coordinate with the electron-donating nitrogen lone pair of the –NH_2_ group, resulting in sluggish desorption kinetics. The prolonged surface residence time of FAM significantly increases the kinetic probability of surface-mediated side reactions, where the nucleophilic primary amine attacks adjacent, surface-bound imine intermediates to yield secondary or tertiary amines. Consequently, accelerating FAM desorption is essential to enhance the yield and selectivity of primary amines.

Hydrogen spillover describes the migration of hydrogen atoms or hydrogen species from metal sites onto the support surface. This phenomenon is particularly important in multi-step tandem reactions as it allows for the spatial separation of hydrogenation active sites from other functional sites while enabling synergistic catalysis through hydrogen migration. In the reductive amination of FAL, an ideal catalyst requires both H_2_ activation sites and N-containing intermediate activation sites. However, introducing Lewis acid sites (*e.g.*, Al^3+^) to activate N-containing intermediates often concurrently inhibits hydrogen spillover capability. To resolve this contradiction, Gao *et al.* designed the Ru/Ga_2_O_3_/MgAlGaO_*x*_ catalyst, innovatively constructing a “dual-scale hydrogen transfer bridge”.^60^ The core of this strategy involves: nanoscale Ga_2_O_3_ coating on Ru nanoparticles to promote initial hydrogen spillover, and atomically dispersed Ga^3+^ in the support acting as secondary spillover channels, working together to facilitate H* migration to widely distributed Al^3+^ Lewis acid active sites (Fig. 2b). This ingenious design allows hydrogen species generated by H_2_ dissociation on Ru sites to efficiently migrate to the vicinity of Al^3+^ sites responsible for activating the Schiff base intermediate, thereby achieving efficient hydrogen transfer and synergistic reaction. Under mild conditions (90 °C, 2 MPa H_2_, low NH_3_ usage), this catalyst exhibited excellent performance with a FAM yield of 93% and a formation rate of 80.43 g_FAM_ g_Ru_^−1^ h^−1^, along with good substrate versatility and recycling stability. H_2_-TPD, WO_3_ colorimetric tests, H-D exchange, and DFT calculations confirmed that the dual-scale Ga structure significantly reduced the hydrogen migration energy barrier and enhanced hydrogen spillover capacity.

Zeolites, with their regular pore structures and tunable acidity, can provide a unique confinement effect when used as catalyst supports, enabling spatial control over reaction selectivity. Liang *et al.* have reported a novel catalyst that “encapsulate” copper active sites within the micropores of MFI-type molecular sieves (Fig. 2d). The medium-sized micropores of MFI act as a “height restriction gate” allowing the correct hydrogenation transition state formed by furfural and hydrogen to pass, while simultaneously squeezing out the larger Piancatelli rearrangement transition state, which requires the participation of a water molecule. This spatially suppresses the occurrence of this side reaction. This “transition-state shape selectively” strategy achieves 100% conversion and 100% selectivity at 70 °C, while maintaining this selective advantage across a broad temperature window of 50∼150 °C.^61^

Metal–support interaction and interface engineering strategies demonstrate that careful design of support properties and interface structures can optimize the spatial configuration of active sites and achieve precise functional division of labor, which is particularly important for complex tandem reactions like reductive amination.

## 4. Precise control of active site structure

Catalyst performance depends not only on chemical composition but also critically on structure at the nano- and even atomic scales. Advances in nano-synthesis and characterization techniques have enabled the precise structural control of active sites, establishing it as a key strategy for enhancing catalytic performance. The exposed facets of metal nanoparticles directly influence surface atom arrangement, coordination environment, and electronic structure, thereby determining catalytic performance. Traditional supported catalysts often yield thermodynamically stable, atomically smooth low-index facets, which are not necessarily the most active for catalytic reactions.

**Fig. 3:**
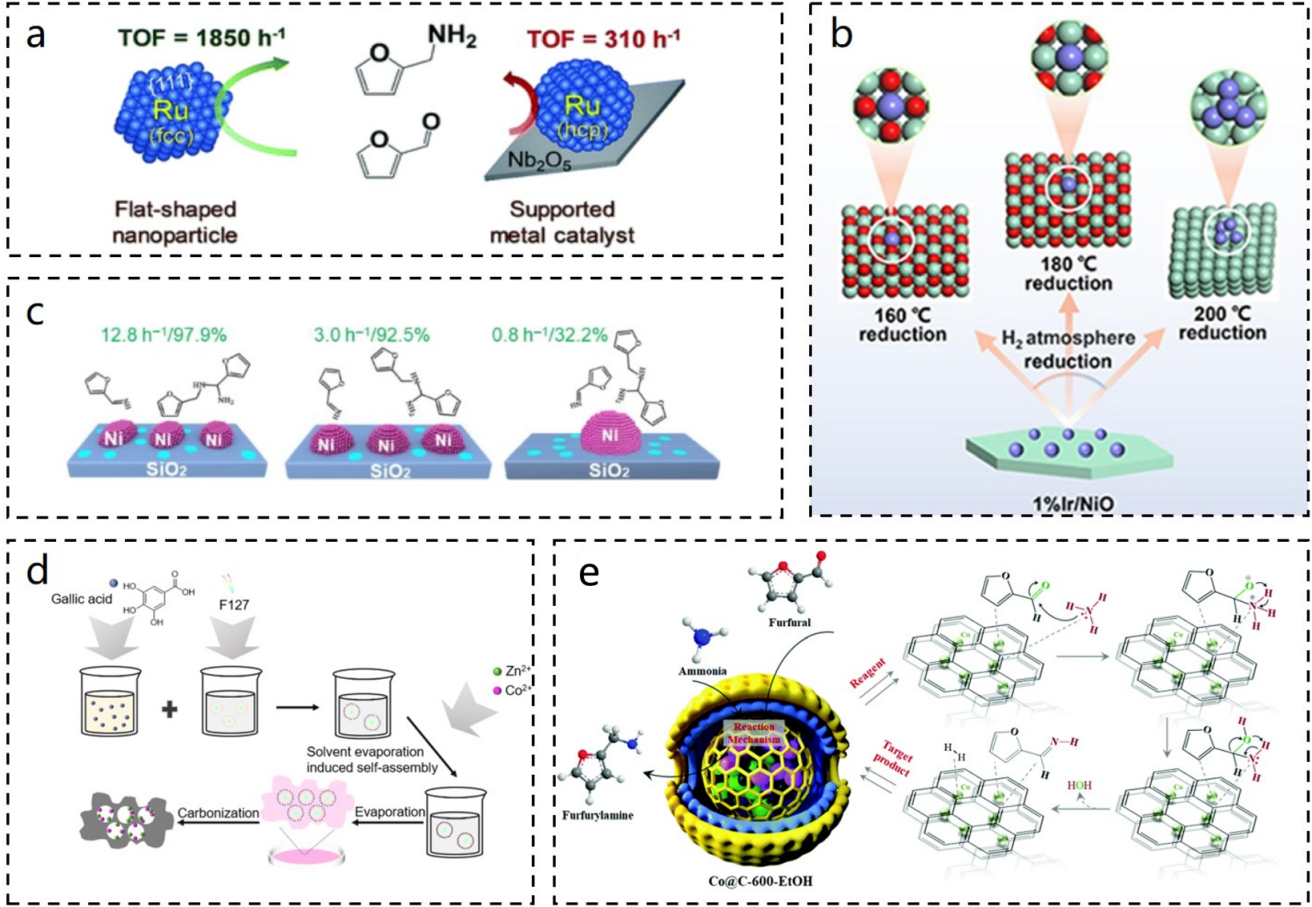
Fine-tuning of active site structure on reductive amination of furfural. (a) Shape-specific ruthenium nanoparticles.^62^ (b) Atomically dispersed Ir catalysts.^64^ Reproduced from ref. 64 with permission from Elsevier, copyright 2023. (c) Robust ultra-small Ni nanoparticles.^63^ Reproduced from ref. 63 with permission from Spring Nature, copyright 2023. (d) CoZn bimetallic catalysts (Co_*x*_Zn_*y*_@MC).^66^ Reproduced from ref. 66 with permission from Royal Society of Chemistry, copyright 2025. (e) Graphene-co-shelled cobalt nanoparticles.^67^ Reproduced from ref. 67 with permission from Royal Society of Chemistry, copyright 2025.

In the study of shape-specific Ru nanoparticles, researchers innovatively used calcium amide (Ca(NH_2_)_2_) as a support.^62^ Through anchoring Ru on its surface and subsequent support removal, they successfully prepared Ru nanoparticles with a flat shape exposing a large proportion of fcc(111) facets. Under condition of 90 °C and 2 MPa H_2_, this catalyst exhibited exceptional performance in the model reaction, with a remarkably high turnover frequency (TOF) of 1850 h^−1^ and a FAM yield of 99%. Mechanistic studies revealed that the high activity of the catalyst are attributed to the atomically rough (111) facets exposed by the flat morphology, while the weak electron-donating character of the metallic Ru^0^ sites facilitated the adsorption and hydrogenation of imine intermediates and suppressed over-hydrogenation or condensation side reactions (Fig. 3a). Similarly, the size and morphology of Ni particles are decisive for their performance in Ni-based catalysts.^63^ Comparing Ni/SiO_2_ catalysts prepared by deposition–precipitation (DP) *versus* impregnation (IM) revealed that the DP-synthesized Ni/SiO_2_-I-DP catalyst featured ultra-small Ni particles (∼2.8 nm), a flat stepped surface structure, and abundant Lewis acid sites. This structure achieved a FAM yield of 97.9% under specific conditions (90 °C, 4 MPa H_2_, 0.8 MPa NH_3_, *tert*-butanol solvent, 1.5 h), with a high reaction rate of 12.8 h^−1^ (Fig. 3c). Mechanistic studies indicated that the small Ni particles provided sufficient hydrogenation active sites for H_2_ activation, while the flat stepped surface and Lewis acid sites synergistically promoted the horizontal adsorption and hydrogenation of intermediates (primary imine and gem-diamine), leading to the highly selective formation of FAM.

Single-Atom Catalysts (SACs), where metal species are anchored as isolated single atoms on a support, maximize atom utilization efficiency and provide uniform active sites, offering an ideal platform for understanding catalysis at the atomic level. In the study of Ir/NiO SACs, researchers precisely tuned the coordination environment of Ir (Ir–O, Ir–Ni, or Ir–Ir) by reducing an Ir precursor supported on NiO nanosheets at different temperatures (160, 180, 200 °C, Fig. 3b).^64^ The optimal catalyst, 1%Ir/NiO-180, achieved complete FAL conversion and 99.2% FAM selectivity in 3 hours at 80 °C under 1 MPa H_2_ with aqueous ammonia. Combined *in situ* FT-IR and DFT calculations suggested that the Ir–Ni interface sites significantly enhanced the adsorption of the Schiff base intermediate and promoted NH_3_ dissociation, efficiently driving the subsequent amination to the target product while suppressing over-hydrogenation. Theoretical studies further reveal the origin of high selectivity in SACs. DFT calculations on the Ru_1_–N_3_/C SAC model indicated that Ru_1_–N_3_/C can moderately co-activate NH_3_ and H_2_, avoiding excessive H_2_ dissociation leading to direct aldehyde hydrogenation. The favored pathway involves FAL dehydrating with NH_3_ to form an imine intermediate, followed by H_2_ hydrogenation to FAM, rather than the furfuryl alcohol pathway. Kinetic analysis showed that the rate constant for the amination path was 5 to 8 orders of magnitude larger than that for the hydrogenation path over Ru_1_–N_3_/C, and low temperature favored suppressing the dimerization of FAM.^65^

Bimetallic catalysts often exhibit performance superior to monometallic ones due to the electronic and geometric effects (ensemble effect) between the two metals. The CoZn@MC bimetallic catalyst was prepared *via* a one-pot solvent evaporation-induced self-assembly (EISA) strategy (Fig. 3d). By tuning the Co/Zn ratio, the Co_1_Zn_3_@MC catalyst achieved complete FAL conversion and a 97.5% FAM yield under mild conditions (100 °C, 1.5 MPa H_2_, 3 h).^66^ Characterization revealed that introducing an appropriate amount of Zn not only promoted high dispersion of Co particles (average size 5.71 nm) and increased the specific surface area (658.4 m^2^ g^−1^) and pore structure but also modulated the electron density of Co through electron transfer from Zn to Co, enhancing its intrinsic catalytic activity. Graphene-encapsulated cobalt nanoparticles (Co@C) utilize a core–shell structure to protect active sites and integrate functions.^67^ This catalyst confines uniform Co nanoparticles within porous graphitic carbon spheres, leveraging the electron penetration effect of the graphene shell to enhance surface catalytic activity. Under optimized conditions (90 °C, 4 h, 2 MPa H_2_, 7 M NH_3_ in MeOH), it showed excellent performance, with >99% conversion and an 86.9% FAM yield (Fig. 3e). Mechanistic analysis suggested metallic Co facilitates C=O bond activation to form the imine intermediate, while surface Lewis acid sites are responsible for hydrogenating the imine to the amine. The graphene shell enhances electron transfer *via* charge redistribution while protecting Co nanoparticles from leaching.

Strategies for precise active site structure control demonstrate that precise manipulation at the nano- and atomic scales can create active sites with specific structures and functions, leading to leaps in catalytic performance. Progress in this field heavily relies on advances in sophisticated synthesis methods and characterization techniques.

## 5. Acid–base site synergistic catalysis and reaction path innovation

Reductive amination is a classic tandem reaction involving the condensation (dehydration) of carbonyl groups with amines and the hydrogenation of imines. Therefore, successful catalysts require both hydrogenation functionality (usually provided by metal sites) and dehydration/activation functionality (often provided by acid–base sites). Rational design of catalyst acidity/basicity and innovation in reaction pathways can significantly improve reaction efficiency and selectivity.^68,69^

### 5.1 Support acidity tuning and frustrated lewis pair (FLP) mechanisms

The acidic properties (type, strength, and number) of the support are crucial for reaction selectivity, as they affect the adsorption and activation of reactants and intermediates.

**Fig. 4:**
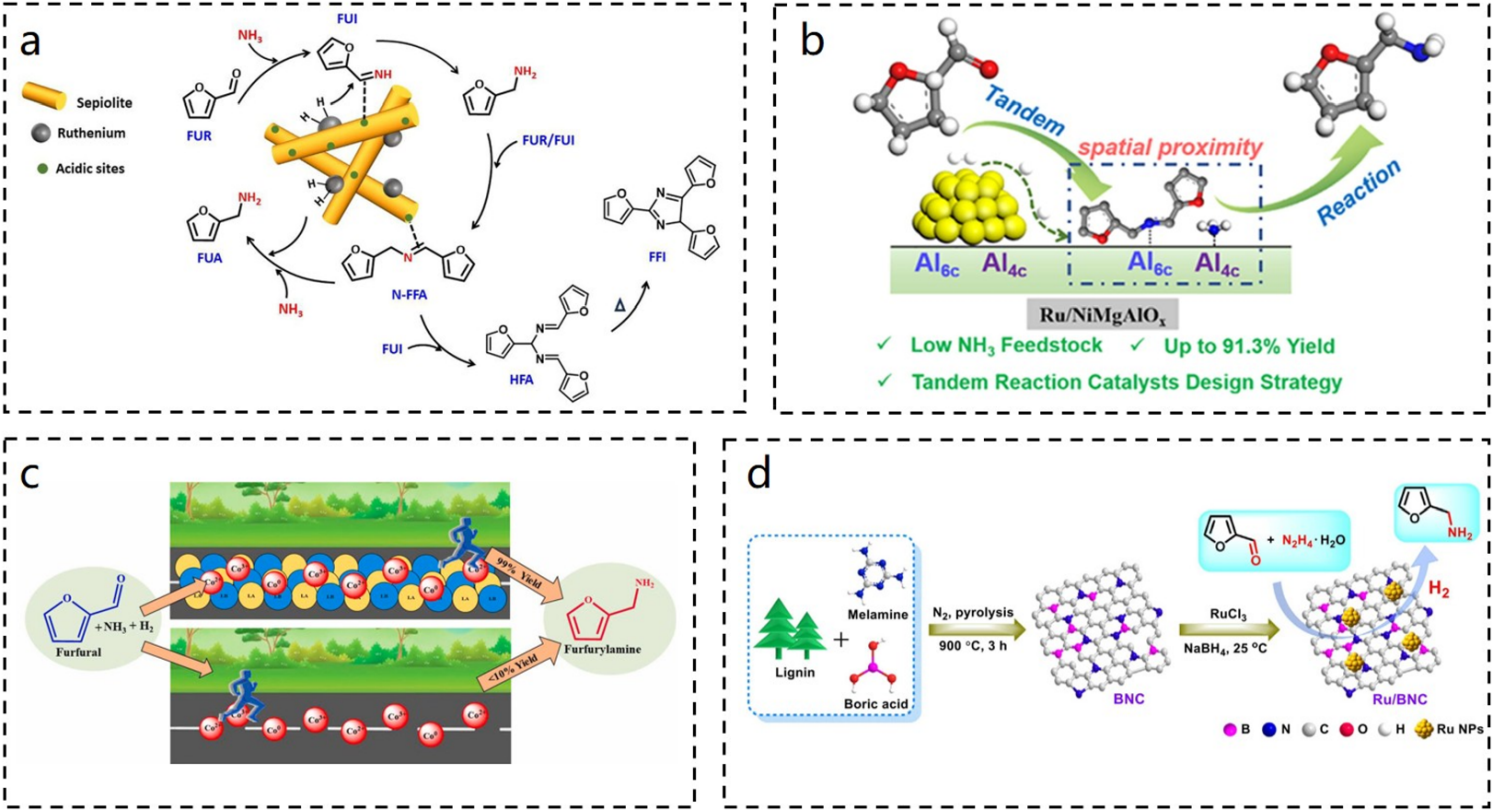
Acid–base site synergistic on reductive amination of furfural. (a) Ru supported on acid-treated sepiolite (Ru/ASep).^70^ Reproduced from ref. 70 with permission from John Wiley and Sons, copyright 2025. (b) Rational regulation of spatially adjacent Al_4c_ and Al_6c_ sites on Ru/NiMgAlO_*x*_ catalysts.^72^ Reproduced from ref. 72 with permission from Elsevier, copyright 2022. (c) Frustrated lewis acid–basic pair in Co/HAP.^73^ Reproduced from ref. 73 with permission from Elsevier, copyright 2025. (d) Frustrated lewis acid–basic pair in Ru/BNC catalysts.^26^ Reproduced from ref. 26 with permission from Elsevier, copyright 2022.

In the Ru/ASep (Ru supported on acid-treated sepiolite) catalyst, acid treatment of the sepiolite support significantly increased the number and strength of Lewis acid sites.^70^ This acidity modification promoted the activation of the C=N bond, increasing the FAM yield dramatically from 43.8% for Ru supported on untreated sepiolite (Ru/Sep) to 98.4%. NH_3_-DRIFT and poisoning experiments (complete reaction stoppage upon poisoning Lewis acid sites with KSCN) confirmed the critical role of Lewis acid sites. Acid treatment not only increased surface Lewis acidity but also improved Ru dispersion and reducibility, enhancing H_2_ activation capability (Fig. 4a). A similar acidity tuning strategy was employed for the Ru/HZSM-5 catalyst.^71^ the strong interaction between Ru and aluminum species in HZSM-5 forms Ru–O–Al bonds, which not only enhances the strong acid sites of the support but also leads to the co-existence of RuO_2_ (providing Lewis acidity) and metallic Ru (responsible for hydrogenation). Studies show that the SiO_2_/Al_2_O_3_ ratio of HZSM-5 significantly affects its acidity and thus the catalytic performance. Under optimized conditions (100 °C, 3 MPa H_2_), a FAM yield of 76% was achieved, and the catalyst could be recycled five times without significant loss of activity. Reaction pathway analysis indicates that the activation of intermediates by acid sites and the activation of H_2_ by metal sites jointly determine reaction selectivity.

A more sophisticated acid site design is exemplified by the Ru/Ni_1_MgAlO_*x*_ catalyst, which successfully addressed the challenge of low selectivity under low ammonia conditions by constructing spatially adjacent and functionally complementary Al_4_c (tetra-coordinated, strong Lewis acid) and Al_6_c (hexa-coordinated, weak Lewis acid) sites.^72^ At a low NH_3_/FAL molar ratio of 3 : 1, it achieved 100% FAL conversion with a 91.3% FAM yield. Mechanistic studies revealed that Al_4_c preferentially adsorbs and activates NH_3_, while Al_6_c adsorbs the Schiff base intermediate. Their spatial proximity and suitable ratio promote the preferential reaction between NH_3_ and the intermediate to form the primary amine (Fig. 4b). Frustrated lewis pairs (FLPs) are active sites in which steric hindrance prevents the complete neutralization of a Lewis acid and a Lewis base. They can heterolytically activate small molecules like H_2_, enabling hydrogenation under mild conditions. The inherent acid–base pairs of the HAP support cooperate with Co^0^ species in the Co/HAP (cobalt supported on hydroxyapatite) catalyst, creating a FLP-like mechanism (Fig. 4c). Studies indicate that Co^0^ species are responsible for the heterolytic dissociation of H_2_ and the hydrogenation of the C=N bond, while the Lewis acid–base sites on HAP synergistically promote the activation of C=O and C=N bonds. This synergy enabled the 7Co/HAP catalyst (7 wt% Co loading) to achieve 99% FAL conversion and >99% FAM selectivity in 0.5 hours at 110 °C and 1 MPa H_2_ in methanol, maintaining excellent performance even at gram-scale.^73^ Ru/BNC (Ru supported on B/N co-doped carbon) is another prominent example of the FLP mechanism (Fig. 4d).^26^ This catalyst utilizes the abundant FLPs on the BNC support surface to synergistically enhance the hydrogen activation capability of Ru sites. Under conditions of 80 °C, 2.0 MPa H_2_, and an N_2_H_4_/FAL molar ratio of 4 : 1, the FAM yield exceeded 99%. Mechanistic studies showed that the B–N FLPs on the BNC surface promote H_2_ heterolysis, while the competitive adsorption of hydrazine moderately inhibits catalyst activity, preventing over-hydrogenation of the product.

### 5.2 Alternative nitrogen sources and reaction path innovation

To circumvent the complex side reaction network associated with using NH_3_ as the nitrogen source, researchers have explored alternative nitrogen sources like hydrazine hydrate (N_2_H_4_·H_2_O) and amines,^74^ thereby altering the reaction pathway and improving selectivity.

The hydrazine route offers the advantage of rapidly forming a stable hydrazone intermediate with FAL, avoiding both direct FAL hydrogenation and homogeneous side reactions with NH_3_. Both Ru/BNC and CoZn@MC catalysts achieved nearly quantitative FAM yields using hydrazine hydrate, demonstrating the superiority of this pathway.^26,66^ Reaction path analysis indicates that FAL first rapidly condenses with hydrazine hydrate to form the hydrazone intermediate, which is then hydrogenated stepwise to the target FAM, effectively avoiding common trimerization side reactions seen in the traditional NH_3_ route.

To eliminate the reliance on high-pressure H_2_, a photocatalytic hydrogen transfer strategy has been developed. Ultrafine Ru nanoclusters (∼0.9 nm) supported on P25 (TiO_2_) utilized ethanol as a hydrogen donor and electron donor under light irradiation at room temperature, achieving 93.5% FAL conversion and 92.9% FAM selectivity.^75^ Mechanistic studies showed that photocatalytic dehydrogenation of ethanol provides active hydrogen, and the imine intermediate is hydrogenated on the Ru cluster surface to form the target amine, with the size effect of the Ru clusters effectively suppressing side reactions.

Strategies involving acid–base site synergy and reaction path innovation show that rational design of catalyst acid–base properties and selection of appropriate reaction pathways can fundamentally alter reaction selectivity and efficiency. This research direction is moving toward greener, milder, and more efficient approaches.

## 6. Future perspectives

Despite significant progress, several challenges remain toward practical industrial application. Future research should focus on the following areas:

(1) Advanced development and stability enhancement of non-noble metal catalysts: while Ni- and Co-based catalysts show great potential, their resistance to ammonia poisoning, metal leaching, and sintering under long-term operation needs improvement. Strategies like novel support design, alloying, and carbon layer encapsulation should be pursued to enhance durability. Exploring new support materials like non-pyridinic N-doped carbons and nitrides (*e.g.*, Mo_2_N) that form strong interactions with non-noble metals could improve stability under harsh reductive amination conditions (ammonia-rich, steam).

(2) Real-time, *In situ* mechanistic elucidation: current understanding of mechanisms often relies on *ex situ* characterization and theoretical calculations. Developing and applying advanced *operando/in situ* techniques (*e.g.*, *in situ* XAS, IR, Raman) to track the evolution of intermediates and the dynamic changes of active sites under real reaction conditions is crucial for revealing the true catalytic cycle and guiding catalyst design. Particular attention should be paid to fundamental questions like the adsorption configuration of intermediates on the catalyst surface, the mode of H_2_ activation (homolytic *vs.* heterolytic), and variations in the rate-determining step with reaction conditions.

(3) Innovation in low-ammonia/ammonia-free reaction systems: using excess ammonia (NH_3_/FAL molar ratios up to 30 : 1) results in low atom economy, equipment corrosion, high energy consumption for ammonia recovery, and environmental concerns. Developing efficient catalyst systems suitable for low-ammonia (*e.g.*, NH_3_/FAL <5 : 1) or even ammonia-free conditions (using amines or hydrazine derivatives) and exploring new amination pathways are important future directions. Bio-chemo coupled catalysis and electrocatalytic amination may offer solutions.

(4) Process intensification and continuous flow process development: most studies are still at the batch reactor level. Developing continuous flow fixed-bed reactor processes for reductive amination would improve process efficiency, safety, and catalyst lifetime, facilitating industrialization. Key issues to address include catalyst mechanical strength, reaction heat management, and efficient product-catalyst separation.

(5) Expansion and integration of catalytic systems: extending efficient catalytic systems to the amination of other biomass platform molecules (*e.g.*, 5-hydroxymethylfurfural, levulinic acid) and exploring integrated strategies for the direct production of N-containing chemicals from raw biomass (*e.g.*, lignocellulose) would significantly enhance the overall economics and sustainability of biorefining. Developing robust catalysts tolerant of impurities in crude biomass feeds (*e.g.*, water, inorganic salts, other oxygenates) is particularly important.

(6) AI-assisted catalyst design and optimization: with the accumulation of extensive experimental and theoretical data, utilizing artificial intelligence (AI) and machine learning (ML) to uncover underlying patterns between catalyst composition, structure, and performance holds promise for accelerating the discovery and optimization of new catalytic materials. This requires establishing unified, standardized databases and developing accurate descriptors and models.

With the continuous refinement of catalyst design theories and innovations in synthesis technologies, efficient and highly selective catalytic amination technologies for furfural and other biomass molecules are poised to play an increasingly important role in sustainable chemical industry, thereby paving the way for a greener, low-carbon future.

## 7. Conclusion

This review has systematically summarized recent advances in heterogeneous catalysts for the selective reductive amination of furfural to furfurylamine, categorizing them based on their primary action mechanisms. Research demonstrates that strategies such as electronic modulation, metal–support interaction and interface engineering, precise control of active site structure, and acid–base site synergy can significantly enhance catalyst activity and selectivity. The core of high performance, whether in noble or non-noble metal systems, lies in achieving a precise balance and synergy between H_2_ dissociation/activation capability, adsorption/activation capability for N-containing intermediates (imines, Schiff bases), and inhibition of undesired reaction pathways.

## Conflicts of interest

The authors declare no competing interests.

## Data Availability

This is a review article. No new primary experimental data were generated or analyzed in this study. All data discussed and summarized herein are available from the original research articles cited in the reference list.
